# Outcomes according to initial and subsequent therapies following intracranial progression in patients with EGFR-mutant lung cancer and brain metastasis

**DOI:** 10.1371/journal.pone.0231546

**Published:** 2020-04-16

**Authors:** Dong-gon Hyun, Chang-Min Choi, Dae Ho Lee, Sang-We Kim, Shinkyo Yoon, Woo Sung Kim, Wonjun Ji, Jae Cheol Lee

**Affiliations:** 1 Department of Pulmonary and Critical Care Medicine, Asan Medical Center, University of Ulsan College of Medicine, Songpa-Gu, Seoul, Republic of Korea; 2 Department of Oncology, Asan Medical Center, University of Ulsan College of Medicine, Songpa-Gu, Seoul, Republic of Korea; George Washington University, UNITED STATES

## Abstract

In patients with epidermal growth factor receptor (EGFR)-mutant non–small-cell lung cancer (NSCLC) with brain metastases, it remains controversial whether the use of EGFR-tyrosine kinase inhibitor (TKI) alone without radiotherapy (RT) is an optimal approach. Here, we investigated the clinical outcomes according to the use of upfront RT as well as the subsequent therapy following intracranial progression. This single-centre retrospective study included a total of 173 patients who were treated with EGFR-TKI alone (TKI alone group) or with upfront whole-brain RT (WBRT) or stereotactic radiosurgery (SRS) followed by EGFR-TKI (RT plus TKI group). Clinical outcomes according to initial and subsequent therapies following intracranial progression were analysed. There was no significant difference in OS according to the use of upfront RT (TKI alone group, 24.5 months vs. WBRT group, 20.0 months vs. SRS group, 17.8 months; P = 0.186). Intracranial progression was found in 35 (32.7%) of 107 patients in the TKI alone group. Among them, 19 patients who received salvage RT had the better prognosis than others [median overall survival (OS); 28.6 vs. 11.2 months; P = 0.041]. In the RT plus TKI group, 12 (18.1%) of the 66 patients experienced intracranial progression and 3 of them received salvage RT (median OS; 37.4 vs. 20.0 months; P = 0.044). In multivariate analysis, upfront WBRT was associated with trends towards a lower probability of intracranial progression, whereas upfront SRS was found to be an independent risk factor for poor OS. In conclusion, using EGFR-TKI alone for brain metastasis in EGFR-mutant lung cancer patients showed outcomes comparable to those using upfront RT followed by EGFR-TKI. Patients who could not receive salvage RT following intracranial progression had the worst survival regardless of the type of initial treatment.

## Introduction

In patients with non–small-cell lung cancer (NSCLC), the incidence of initial brain metastases at the time of lung adenocarcinoma diagnosis is approximately 20% [1]; furthermore, patients with brain metastases have poor outcomes compared with those without brain metastases [2]. Although radiotherapy (RT) or surgical resection has been the conventional treatment for brain metastases, patient survival rate remains unsatisfactory and severe deterioration of general condition has often been observed owing to neurotoxicity after RT [3,4]. However, the median overall survival (OS) has recently been increasing in patients with epidermal growth factor receptor (EGFR)-mutant lung adenocarcinoma and brain metastases due to the introduction of targeted therapy [5].

Although EGFR-tyrosine kinase inhibitor (TKI) has low cerebrospinal fluid penetration rates [6], it may result in good intracranial response rates due to a high sensitivity of EGFR-mutant tumour to EGFR-TKI [7]. Therefore, upfront EGFR-TKI alone without local RT has been used [8–11] with the advantage of avoiding radiation-induced neurotoxicity until tumour progression [12,13]. However, several studies have shown that upfront RT plus EGFR-TKI could produce a favourable outcome [14,15]. Furthermore, a recent multi-institutional retrospective analysis has revealed that stereotactic radiosurgery (SRS) followed by EGFR-TKI is associated with the longest OS [16]. Thus, proper management of EGFR-mutant NSCLC with brain metastases remains controversial.

Most studies have focused on outcomes according to the presence or absence of RT in initial treatment [14,16]. Hence, it is difficult to find data on the progression pattern after initial treatment and the effects of the subsequent treatments. To determine the optimal management of patients with EGFR-mutant NSCLC with brain metastases, this study investigated the clinical outcomes according to the use of upfront RT (WBRT or SRS) as well as the disease progression pattern and subsequent therapy following intracranial progression.

## Material and methods

### Study design and patients

This retrospective study included patients who were initially diagnosed with EGFR-mutant lung adenocarcinoma and brain metastases between 1st January 2011 and 31st December 2016. Data were collected from patients’ medical records. Inclusion criteria were as follows: 1) patients pathologically diagnosed with EGFR-mutant lung adenocarcinoma; 2) brain metastases confirmed using magnetic resonance imaging (MRI) or computed tomography (CT) scan at the time of initial diagnosis; 3) patients who received EGFR-TKI therapy with or without RT. Patients were excluded if they reported prior EGFR-TKI use, received conventional chemotherapy, underwent surgical resection or brain RT to the lung cancer before study enrolment or had an EGFR-TKI–resistant mutation. Patients were treated with EGFR-TKI alone (TKI alone group) or with upfront whole-brain RT (WBRT) or stereotactic radiosurgery (SRS) followed by EGFR-TKI (RT plus TKI group). This study was approved by the Institutional Review Board (IRB) of Asan Medical Center (IRB No. 2018–0240) and performed in accordance with the amended Declaration of Helsinki. Because this study was the retrospective analysis, IRB confirmed the requirement for informed consent was waived.

### Data variables and response assessment

The following variables were collected for analysis: age, sex, TNM stage (8th edition) [17], smoking history, EGFR mutation, Eastern Cooperative Oncology Group (ECOG) performance status at the time of initial diagnosis, number of brain metastases, size of the largest brain metastasis, presence or absence of extracranial metastases, symptoms associated with brain metastases, type of RT, pattern of progression (intracranial progression vs. systemic) and presence or absence of salvage RT after intracranial progression. EGFR mutations were detected using polymerase chain reaction amplification from the paraffin-embedded tumour samples.

Tumour response was evaluated according to the Response Evaluation Criteria in Solid Tumours, version 1.1, using MR images of the brain; CT scans of the chest, abdomen and pelvis and positron emission tomography images. Objective response rate was defined as complete response (CR; the best tumour response) and partial response (PR), and disease control rate was defined as the sum of CR, PR and stable disease.

### Statistical methods

All the available patient data were compared using χ^2^ test or Fisher’s exact test for categorical variables. OS was calculated from the date of lung adenocarcinoma diagnosis until the date of death due to any cause. Progression-free survival (PFS) was defined as the duration from the date of lung adenocarcinoma diagnosis until the date of growth of a previous lesion, development of a new lesion, death without documented progression or last follow-up. The primary outcome of this study was the comparison of OS in patients according to the type of initial treatment (EGFR-TKI alone, WBRT followed by EGFR-TKI and SRS followed by EGFR-TKI) or the presence of salvage RT. The secondary outcome included PFS, response rate and risk factors associated with death. Kaplan–Meier analysis was used to estimate OS and PFS, whereas log-rank test was used to test the significance of differences. Furthermore, cumulative incidence curves were generated for intracranial progression. Univariable and multivariable Cox proportional hazards regression models identified risk factors associated with death and intracranial progression. A final model was constructed using a stepwise method with backward selection; *p* values < 0.15 in univariate analysis were set for the entry of variables. Two-sided *p* values < 0.05 were considered to indicate significance. All analyses were performed using SPSS ver. 24.0 (IBM Corporation, USA) software.

## Results

### Patient characteristics

We identified 173 eligible patients after applying the exclusion criteria. Of the 173 patients, 107 (61.8%) received EGFR-TKI alone, 36 (20.8%) were treated with WBRT followed by EGFR-TKI and 30 (17.3%) were treated with SRS followed by EGFR-TKI. All of 173 patients were evaluated by MRI scan for CNS metastasis at the time of initial diagnosis. Patient characteristics are shown in Table 1. There were no differences between the TKI alone and RT plus TKI groups regarding age, sex, ECOG performance status, smoking status, number of brain metastases, extracranial metastases at the time of brain metastases, EGFR mutation and EGFR-TKIs. Patients treated with EGFR-TKI alone were more likely to have a N2–3 (51.4% TKI alone vs. 31.8% upfront RT; P = 0.018) and M1a (18.7% TKI alone vs. 0% upfront RT; P < 0.0001) stage tumour at diagnosis. The TKI alone group was less likely to have symptomatic brain metastases (8.4% TKI alone vs. 37.9% upfront RT; P < 0.0001) and largest brain metastases measuring ≤1cm (67.3% TKI alone vs. 25.8% upfront RT; P < 0.0001). In the RT plus TKI group, the number of patients with ≥5 brain metastases was higher in the upfront WBRT group (77.8%) than in the upfront SRS group (6.7%).

**Table 1:** Patient characteristics.

	TKI alone		RT plus TKI (n = 66)		
Characteristic, No. (%)	(n = 107)	*p*-value	WBRT (n = 36)	SRS (n = 30)	*p*-value
Sex					
Male	37 (34.6)	.402	11 (30.6)	16 (53.3)	.061
Female	70 (65.4)		25 (69.4)	14 (46.7)	
Age (years)					
<65	65 (60.7)	.162	29 (80.6)	18 (60.0)	.066
≥65	42 (39.3)		7 (19.4)	12 (40.0)	
T stage at diagnosis					
T1–3	69 (64.5)	.181	21 (58.3)	28 (93.3)	.001
T4	38 (35.5)		15 (41.7)	2 (6.7)	
N stage at diagnosis					
N0–1	52 (48.6)	.018	23 (63.9)	22 (73.3)	.412
N2–3	55 (51.4)		13 (36.1)	8 (26.7)	
M1a stage at diagnosis	20 (18.7)	< .0001	0 (0.0)	0 (0.0)	
ECOG		.251			
0–1	77 (72.0)		20 (55.6)	22 (73.3)	.135
2–4	30 (28.0)		16 (44.4)	8 (26.7)	
Smoking status					
Current/former	36 (33.6)	.505	10 (27.8)	9 (30.0)	.843
Never	71 (66.4)		26 (72.2)	21 (70.0)	
Symptomatic brain metastases					
No	98 (91.6)	< .0001	22 (61.1)	21 (70.0)	.853
Yes	9 (8.4)		14 (38.9)	11 (36.7)	
No. of brain metastases					
1	24 (22.4)	.791*	3 (8.3)	10 (33.3)	< .0001
2–4	30 (28.0)		5 (13.9)	18 (60.0)	
5–9	23 (21.5)		10 (27.8)	2 (6.7)	
≥10*	30 (28.1)		18 (50.0)	0 (0.0)	
Size of largest brain metastases					
≤1 cm	72 (67.3)	< .0001*	10 (27.8)	7 (23.3)	.574*
>1 cm	35 (32.7)		26 (72.2)	23 (76.6)	
Extracranial metastases at time of brain metastases					
No	24 (22.4)	.713	8 (22.2)	9 (30.0)	.575
Yes	83 (77.6)		28 (77.8)	21 (70.0)	
EGFR mutation					
Exon 18	4 (3.7)	.573*	1 (2.8)	3 (10.0)	.429*
Exon 19	68 (63.6)		23 (63.9)	17 (56.6)	
Exon 21	35 (32.7)		12 (33.3)	10 (33.3)	
EGFR-TKIs					
Gefitinib	77 (72.0%)	.239	27 (75.0%)	21 (70.0%)	.078*
Erlotinib	16 (15.0%)		5 (13.9%)	9 (30.0%)	
Afatinib	14 (13.1%)		4 (11.1%)	0 (0.0%)	

* Fisher’s exact test.

**Abbreviations:** ECOG; Eastern Cooperative Oncology Group. EGFR; Epidermal growth factor receptor. TKI; Tyrosine kinase inhibitor. RT; Radiotherapy. WBRT; Whole-brain radiotherapy. SRS; Stereotactic radiosurgery

### Disease progression pattern and treatment outcomes

**Fig 1:**
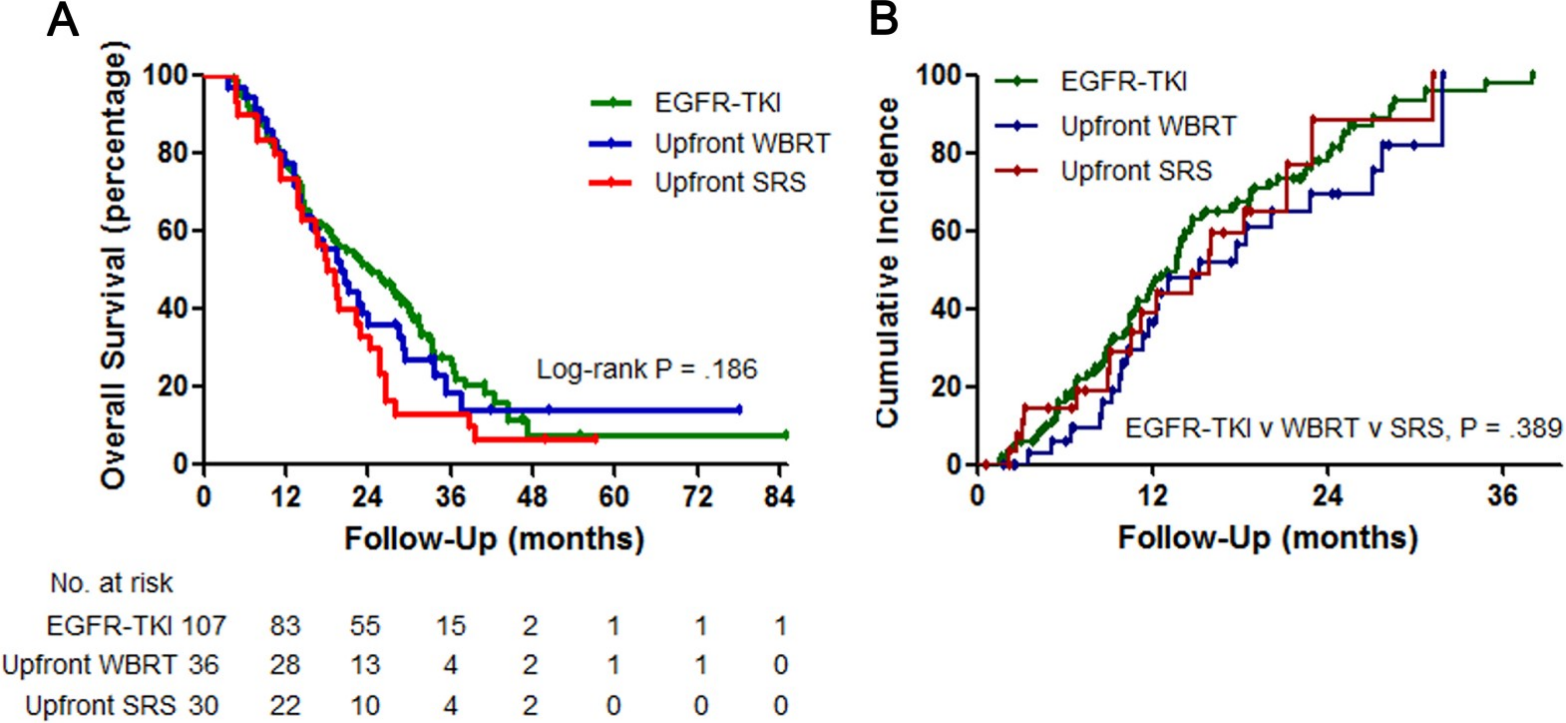
(A) Kaplan–Meier analysis comparing overall survival. Patients treated with epidermal growth factor receptor-tyrosine kinase inhibitor (EGFR-TKI), upfront whole-brain radiotherapy (WBRT) and upfront stereotactic radiosurgery (SRS). (B) Cumulative incidence of intracranial progression. Patients treated with EGFR-TKI, upfront WBRT and upfront SRS.

**Fig 2:**
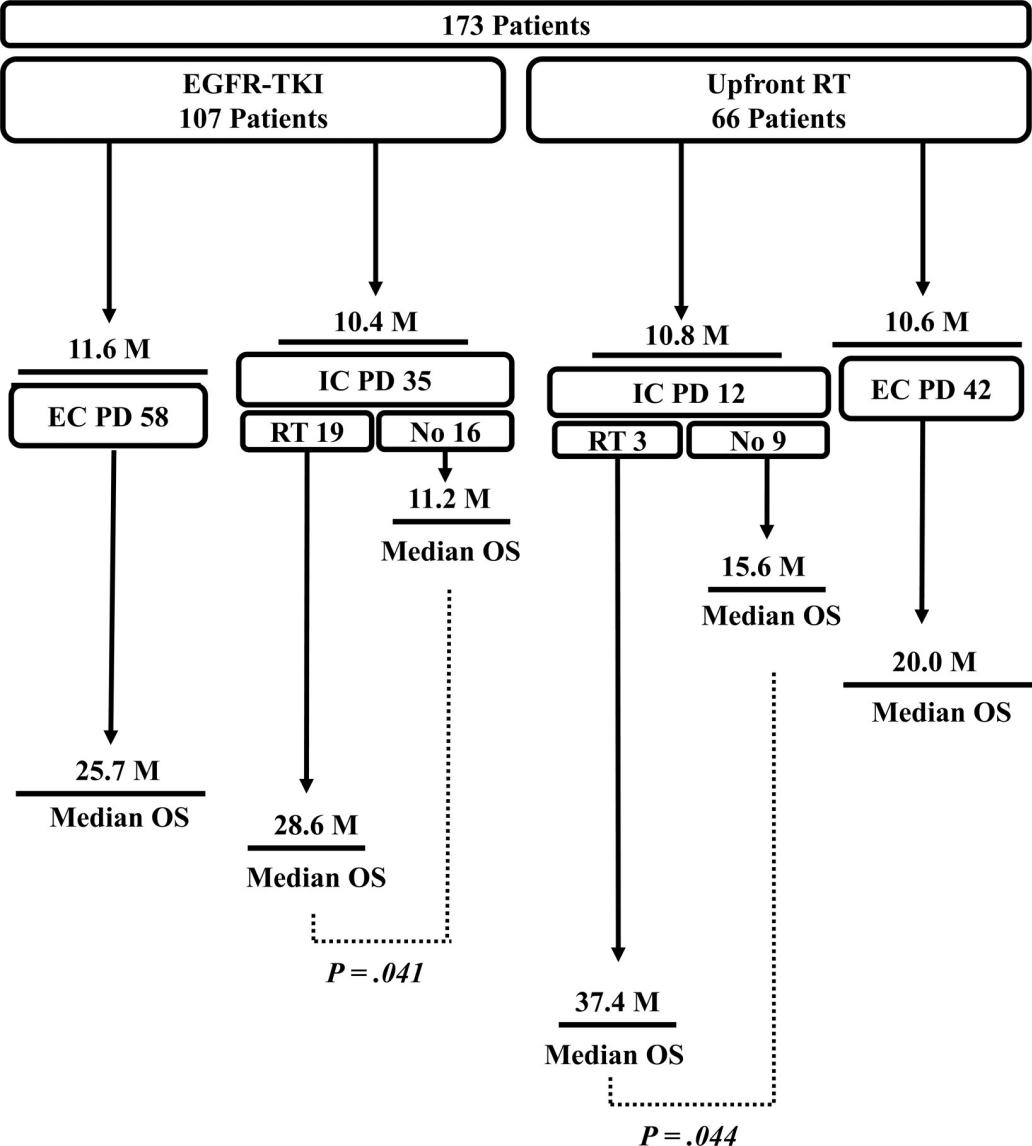
Flow chart describing the course of disease progression and death of all patients. EGFR; Epidermal growth factor receptor. TKI; Tyrosine kinase inhibitor. RT; Radiotherapy. IC; Intracranial. EC; Extracranial. PD; Progression disease. OS; Overall survival. M; Months.

**Fig 3:**
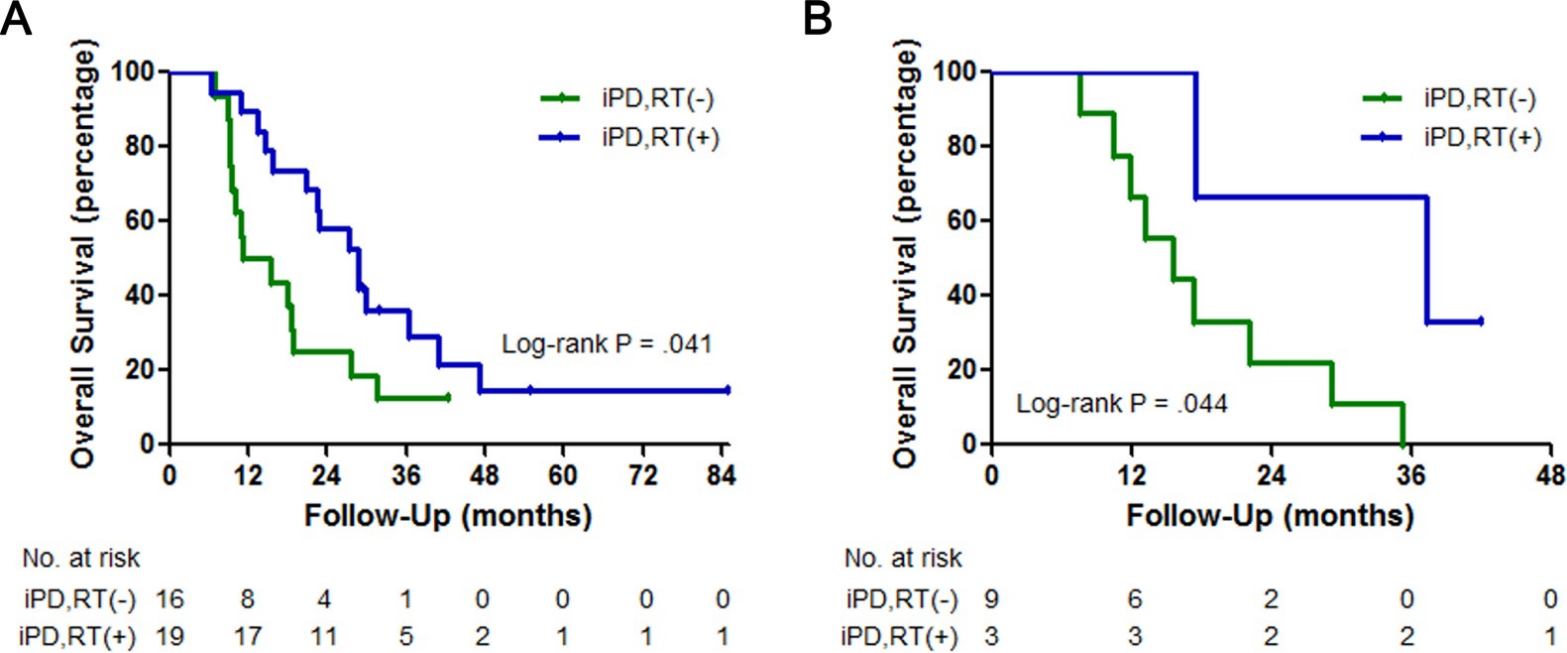
(A) Kaplan–Meier analysis comparing overall survival in initially intracranial progression group of patients treated with epidermal growth factor receptor-tyrosine kinase inhibitor, stratified by salvage radiotherapy (RT). (B) Kaplan–Meier analysis comparing overall survival in initially intracranial progression group of patients treated with upfront RT, stratified by salvage RT. iPD; Intracranial progression disease.

The median follow-up duration was 18.7 (range 1.6–76.8) months. The median count of CNS imaging after initial treatment was 3.0 (range 1.0–24.0) and the median first, second interval between response evaluations was 3.4 (range 0.6–28.6), 2.8 (range 0.0–37.0) months. The median OS for the TKI alone, WBRT and SRS groups was 24.5 months (95% CI, 18.1–30.8), 20.0 months (95% CI, 14.6–25.4) and 17.8 months (95% CI, 13.9–21.8), respectively (log-rank P = 0.186; Fig 1). The median time to intracranial progression for the TKI alone, WBRT and SRS groups was 13.6 months (95% CI, 11.6–15.5), 15.3 months (95% CI, 7.1–23.5) and 15.8 months (95% CI, 10.6–21.0), respectively (log-rank P = 0.389). Intracranial progression developed in 35 (32.7%) of the 107 patients who received EGFR-TKI alone, whereas it developed in 12 (18.1%) of the 66 patients who were treated with upfront RT followed by EGFR-TKI (P = 0.036) (Fig 2). Similar intracranial PFS (10.4 months vs. 10.8 months; P = 0.945) and extracranial PFS (11.6 months vs. 10.6 months; P = 0.754) were observed between patients treated with TKI alone and those who received RT plus TKI. The median OS for patients who received EGFR-TKI alone without and with salvage RT was 11.2 months (95% CI, 2.1–20.4) and 28.6 months (95% CI, 19.8 to 37.4), respectively (log-rank P = 0.041; Fig 3). Furthermore, there was a significant difference in OS according to the presence or absence of subsequent RT in the RT plus TKI group (37.4 months; 95% CI, 5.6–69.2 vs. 15.6 months; 95% CI, 8.6–22.6) (log-rank P = 0.044). Most patients without salvage RT could not undergo RT due to their deteriorating general condition. Regarding intracranial response at 1st evaluation, no significant difference in the intracranial objective response rate was observed among the three groups (TKI alone group, 62.6% vs. WBRT group, 72.2% vs. SRS group, 60.0%; P = 0.508) (Table 2). However, intracranial disease control rate was higher in the RT plus TKI group than in the TKI alone group (WBRT group, 94.4% vs. SRS group, 83.3% vs. TKI alone group, 72.0%; P = 0.014).

**Table 2:** Comparison of the response among the three groups at 1st evaluation after treatment.

Variable, No. (%)	EGFR-TKI (n = 107)	Upfront WBRT (n = 36)	Upfront SRS (n = 30)	*p*-value
Intracranial Response				< .0001*
CR	20 (18.7)	0 (0.0)	0 (0.0)	
PR	47 (43.9)	26 (72.2)	18 (60.0)	
SD	10 (9.3)	8 (22.2)	7 (23.3)	
PD	30 (28.0)	2 (5.6)	5 (16.7)	
Intracranial ORR	67 (62.6)	26 (72.2)	18 (60.0)	.508
Intracranial DCR	77 (72.0)	34 (94.4)	25 (83.3)	.014

* Fisher’s exact test.

**Abbreviations:** EGFR; Epidermal growth factor receptor. TKI; Tyrosine kinase inhibitor. WBRT; Whole-brain radiotherapy. SRS; Stereotactic radiosurgery. CR; Complete response. PR; Partial response. SD; Stable disease. PD; Progression disease. ORR; Objective response rate. DCR; Disease control rate.

### Prognostic factors

Multivariable model controlling for significant covariables such as age, ECOG performance status, upfront WBRT or SRS, smoking status, salvage RT and extracranial metastases at the time of brain metastases revealed that upfront SRS (HR 1.67; 95% CI, 1.08–2.58; P = 0.022), age < 65 years (HR 0.60; 95% CI, 0.42–0.84, P = 0.004), ECOG performance status 2–4 (HR 1.80; 95% CI, 1.24–2.61, P = 0.002) and extracranial metastases at time of brain metastases (HR 2.01; 95% CI, 1.27 to 3.17; P = 0.003) were independent risk factors for predicting OS (Table 3). Upfront WBRT was also associated with trends towards a lower probability of intracranial progression (HR 0.55; 95% CI, 0.33–0.91; P = 0.021). However, other variables such as sex, EGFR mutation and number of intracranial metastases did not influence OS and intracranial PFS.

**Table 3:** Multivariable model of risk factors to predict intracranial progression-free survival and overall survival.

	Progression-free survival			Overall survival		
Variable	HR	95% CI	*p*-value	HR	95% CI	*p-*value
Upfront WBRT v EGFR-TKI	0.55	0.33 to 0.91	.021	1.08	0.70 to 1.67	.723
Upfront SRS v EGFR-TKI	0.95	0.56 to 1.60	.832	1.67	1.08 to 2.58	.022
Age (years)						
<65 vs. ≥65				0.60	0.42 to 0.84	.004
ECOG performance status						
2–4 vs. 0–1	1.92	1.28 to 2.90	.002	1.80	1.24 to 2.61	.002
Smoking status						
Yes vs. No				1.40	0.98 to 2.02	.064
Extracranial metastases at time of brain metastases						
Yes vs. No	1.49	0.95 to 2.31	.080	2.01	1.27 to 3.17	.003

**Abbreviations:** EGFR; Epidermal growth factor receptor. TKI; Tyrosine kinase inhibitor. RT; Radiotherapy. WBRT; Whole-brain radiotherapy. SRS; Stereotactic radiosurgery. ECOG; Eastern Cooperative Oncology Group. HR; Hazard ratio. CI; confidence interval.

## Discussion

In this study, the use of upfront RT was not associated with a better prognosis than treatment with EGFR-TKI alone, although patients who received upfront RT followed by EGFR-TKI had a high disease control rate at the initial evaluation of treatment response. In addition, among patients who developed intracranial progression, OS improved in patients in whom salvage RT was applied to the intracranial lesions compared with that in patients without salvage RT.

Several mechanisms have been proposed for the progression of intracranial lesions. First, resistant mutations against TKI resistance could induce intracranial progression [18]. The p.Thr790Met point mutation (T790M) in the gene encoding EGFR is the most common cause of TKI resistance in lung cancer; it can reduce binding of TKIs to EGFR [19]. *MET* gene amplification [20] and transformation into small-cell lung cancer [21] are also associated with TKI resistance. Concurrent progression of both intracranial and extracranial lesions may be caused by these mechanisms, whereas only intracranial progression would occur due to different causes [22,23]. The altered penetration of TKI into the central nervous system across the blood–brain barrier has been considered as the one of mechanisms [24]. A decreased concentration of TKI in cerebrospinal fluid provokes the reduction of TKI-mediated inhibition of downstream signalling on the intracranial lesions, causing intracranial progression [22]. Through a similar mechanism, increased p-glycoprotein may lead to intracranial progression via a decrease in intracranial TKI concentration [25]. There were many patients with intracranial progression in this study; in them, the progression was likely related to a change in the intracerebral TKI concentration.

In the present study, no significant differences in intracranial progression and survival benefit were observed between patients treated with EGFR-TKI alone and those who received upfront RT followed by EGFR-TKI. Because there were significantly more patients who had large and symptomatic brain metastases in the RT plus TKI group than in the TKI alone group, these results must be carefully interpreted. Moreover, approximately half the patients in the TKI alone group received salvage RT, but only a few patients in the RT plus TKI group received it. This difference might have influenced the clinical outcomes because an analysis of patients who had intracranial progression showed that patients who received salvage RT had a longer median OS than those who did not, regardless of the initial treatment. The third-generation EGFR-TKI commonly used nowadays can be considered as a treatment option instead of salvage RT because of its much better intracranial effect than that of previous EGFR-TKIs [26]. However, it is very difficult to obtain brain tissues for the identification of T790M in patients with intracranial progression. Although a liquid biopsy using a blood sample recently has been introduced into the test for T790M, the result through liquid biopsy occasionally seems to show discrepancy of T790M expression between actual tissue sample and blood sample. Therefore, salvage RT is more likely chosen to manage intracranial progression in real practice. Further study will be needed for EGFR mutant NSCLC patients with initially brain metastasis who received the third generation TKIs as the first line treatment to investigate the clinical outcomes according to the use of upfront RT as well as the salvage RT following intracranial progression. Because there could be a selection bias that patients without salvage RT might have poorer performance than those with salvage RT, further prospective randomised trials are needed to investigate the effect of salvage RT in patients with intracranial progression.

The clinical outcomes in this study seem to be consistent with those reported by prior studies evaluating the use of EGFR-TKI alone or of upfront RT followed by EGFR-TKI [16,27–35]. Although the disease control rate after the 1st treatment was higher in RT plus TKI groups than in the TKI alone group, the median OS of patients who received EGFR-TKI alone, upfront WBRT and upfront SRS in the present study was 24.5, 20.2 and 18.5 months, respectively, showing no significant differences. In two studies conducted in South Korea, the use of upfront RT did not provide more beneficial outcomes than the use of EGFR-TKI alone [27,33]. Other retrospective studies have also reported similar outcomes, with OS ranging from 21.6 months to 31.9 months according to the type of treatment, despite the higher intracranial PFS with upfront RT [30–32]. In addition, a meta-analysis that analysed the 5 studies for the comparison of WBRT plus EGFR-TKIs and EGFR-TKIs alone in Cerebral metastatic NSCLC patients has presented that EGFR-TKIs provides similar clinical outcomes compared with WBRT plus EGFR-TKIs [35]. These studies are likely to support the non-inferiority of EGFR-TKIs alone treatment. Conversely, a retrospective multi-institutional analysis has shown a better prognosis than that observed in the present study; in that study, the median OS of patients treated with SRS followed by EGFR-TKI, WBRT followed by EGFR-TKI and EGFR-TKI followed by SRS or WBRT was 47, 31 and 25 months, respectively [16]. We believe that accumulating more data in the near future can help explain this issue.

This study has several limitations. First, it was a single-centre retrospective study, which would lead to all of the inherent biases in such a study. There were significantly different baseline characteristics between the groups. The RT plus TKI group had more patients with CNS symptom and > 1cm size of brain metastasis than the TKI alone group, which might have resulted in better prognostic effects on patients in the TKI alone group. However, a tumor burden such as N2-3 and M1a of the TKI alone group was larger than the RT plus TKI group. Considering these factors, the results that there were no significant differences from clinical outcomes between the two groups in rear world practice could be reasonable and meaningful. In addition, this study had the homogeneity of the patient population with confounding factors including age, ECOG to affect the clinical outcomes and we also tried to minimize bias by using multivariate model. Further prospective studies with well-balanced cohort are needed in the future. Second, the evaluation of intracranial disease using brain imaging was not regularly performed. Approximately 40% patients received CNS evaluation only 1 time, while some patient was evaluated by maximal 24 times for CNS metastasis. A differential bias might have resulted from missing asymptomatic brain metastases. Nevertheless, most patients in this study were evaluated for CNS metastasis every approximately three months. Also, the strength of this study is that we analysed all patients’ pattern of cancer progression and subsequent treatment course according to the progression pattern. Third, we did not account for several RT-induced neurotoxicities, which is one of the most important reasons for the deterioration of patients’ quality of life. Despite of this missing data, it is important to note that we evaluated the impact of salvage RT, the statistically significant finding, on the final outcome. Considering these limitations, the findings in this study need to be validated in prospective trials with large scale.

## Conclusions

Although EGFR-TKI alone for brain metastasis in EGFR-mutant lung cancer showed outcomes comparable to those obtained with upfront RT followed by EGFR-TKI, a proportion of patients who could not receive RT following intracranial progression had the worst survival in this study. In patients with only intracranial progression, salvage RT to the intracranial lesions might have survival benefits. Further prospective studies are needed to determine the optimal management of patients with EGFR-mutant NSCLC who initially develop brain metastases.
